# Supplementary figures and images for: Nifuroxazide suppresses PD-L1 expression and enhances the efficacy of radiotherapy in hepatocellular carcinoma
Source: eLife. 2024 Mar 5;12:RP90911. doi: 10.7554/eLife.90911 (PMC10942647; doi:10.7554/eLife.90911)

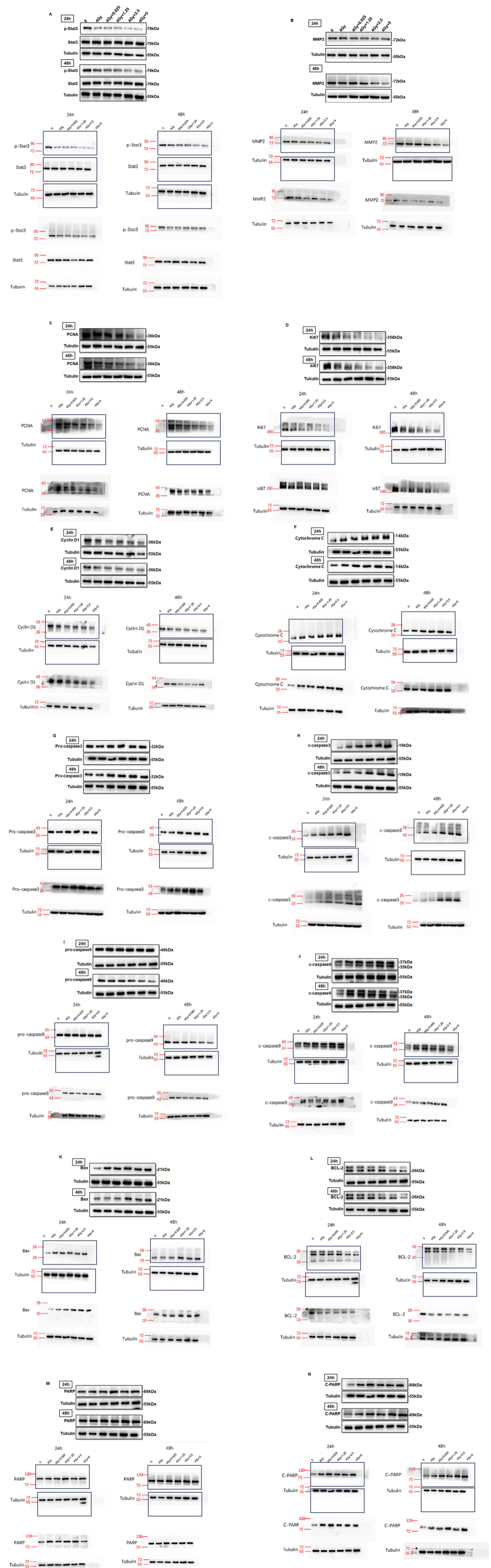

Figure 2

Supplement: Figure 2—source data 4. [file elife-90911-fig2-data4.pdf]

E

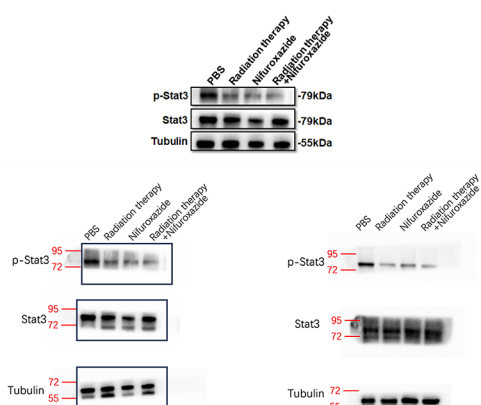

G

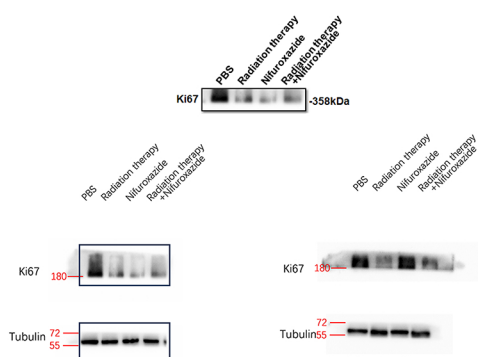

I

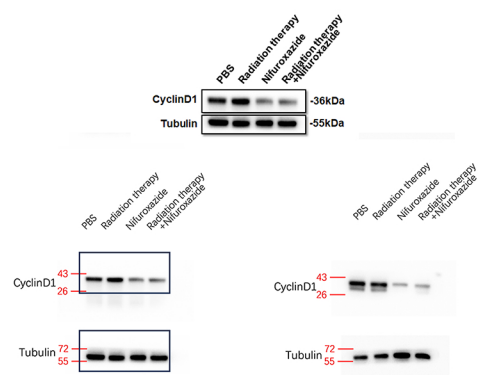

K

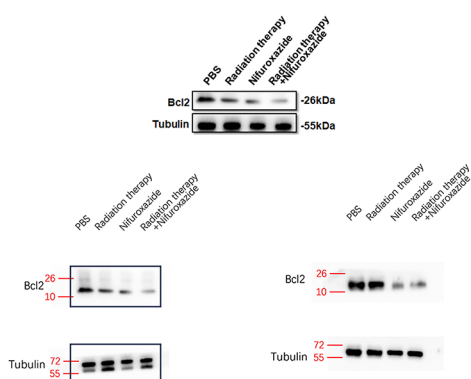

L

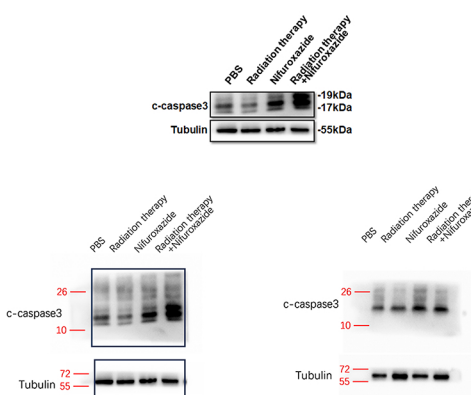

F

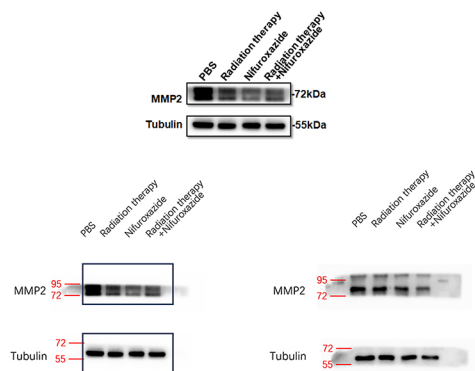

H

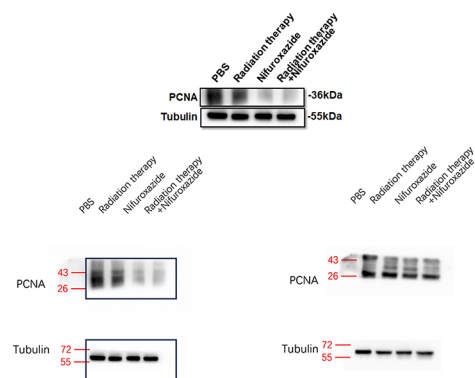

J

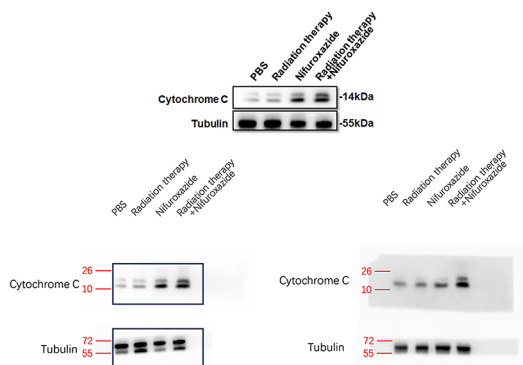

M

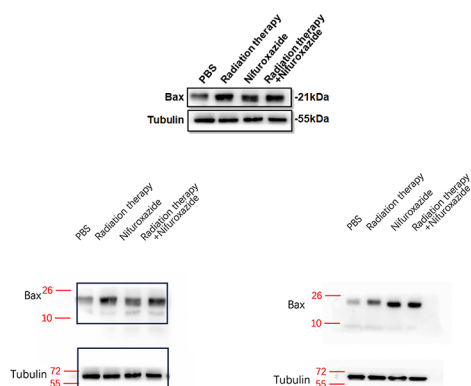

N

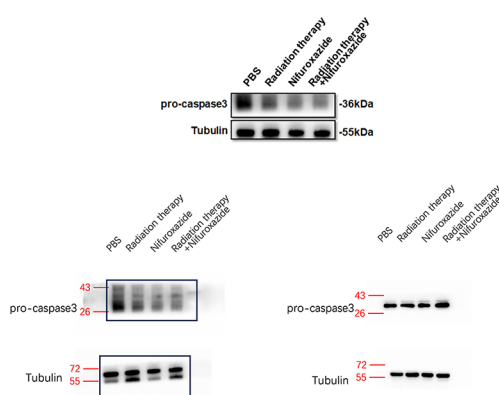

Figure 4

Supplement: Figure 4—source data 2. [file elife-90911-fig4-data2.pdf]

F

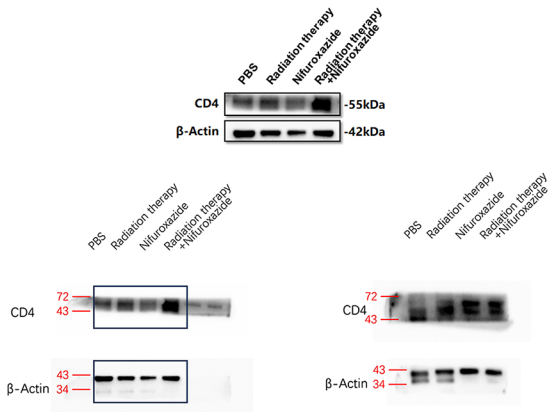

G

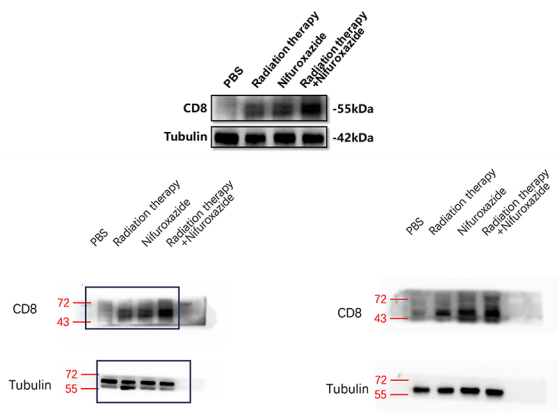

H

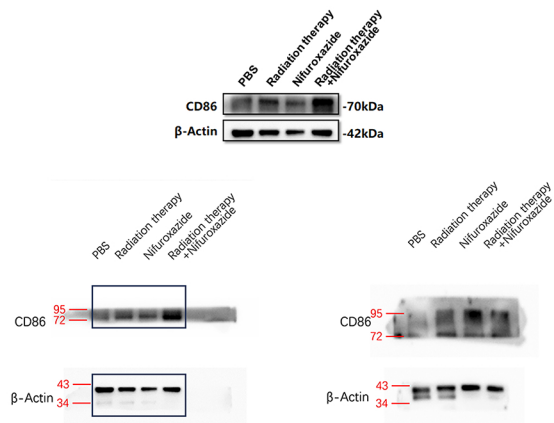

I

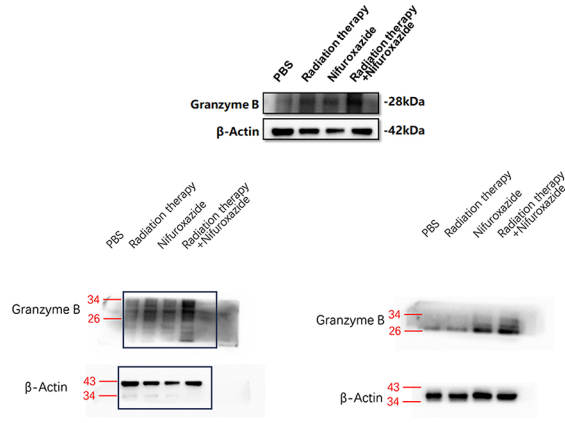

Figure 5

Supplement: Figure 5—source data 2. [file elife-90911-fig5-data2.pdf]

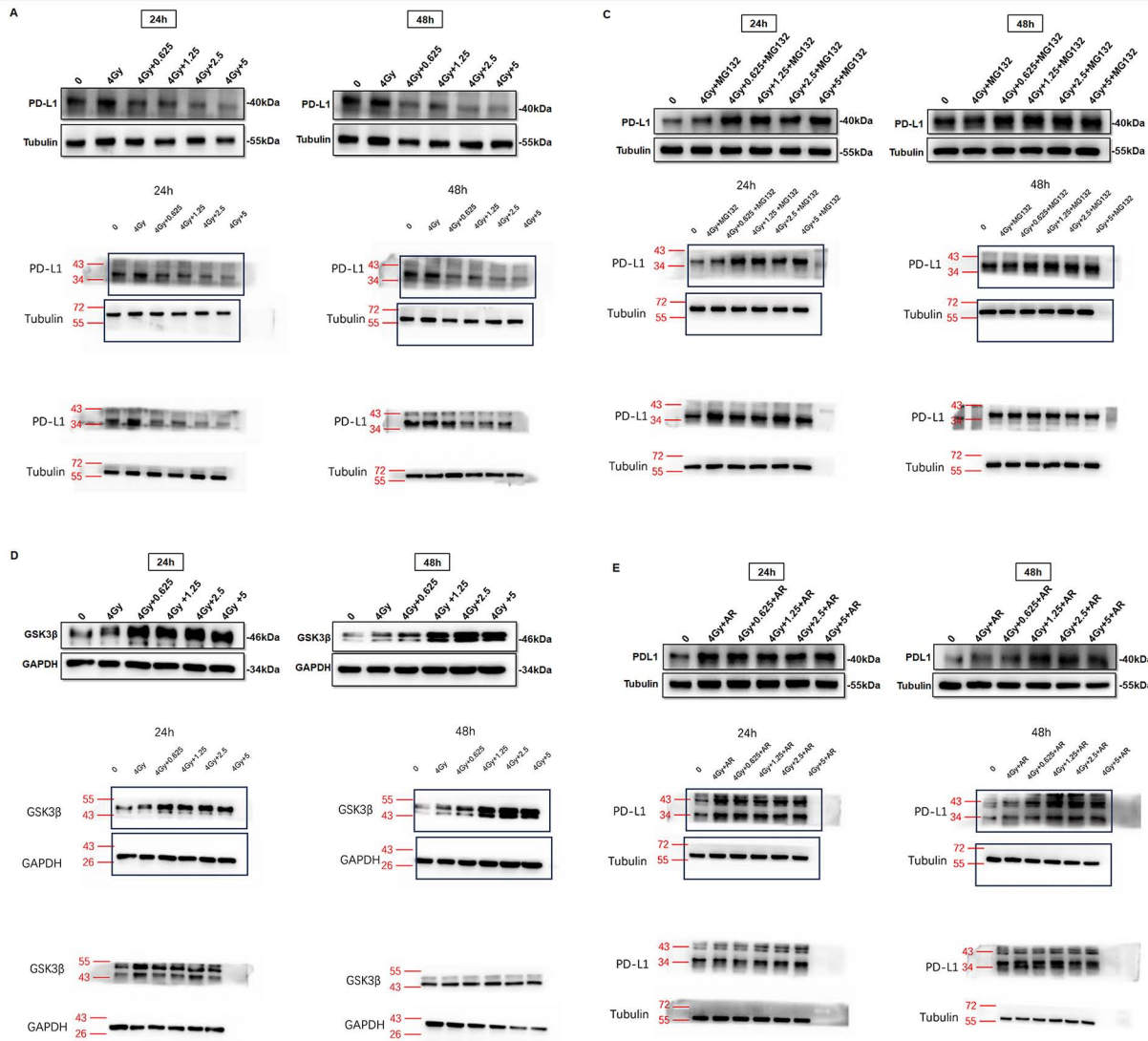

**Figure 7**

Supplement: Figure 7—source data 2. [file elife-90911-fig7-data2.pdf]

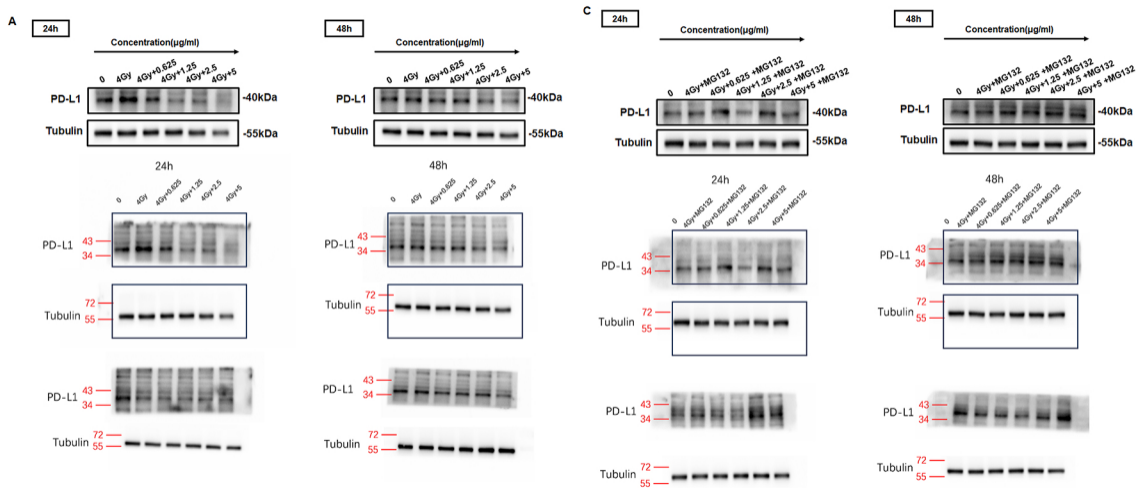

**Supplementary Figure 3**

Supplement: Figure 7—figure supplement 1—source data 2. [file elife-90911-fig7-figsupp1-data2.pdf]

C

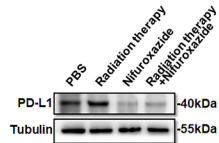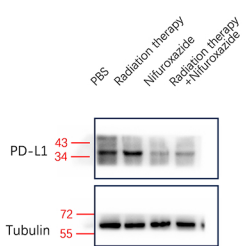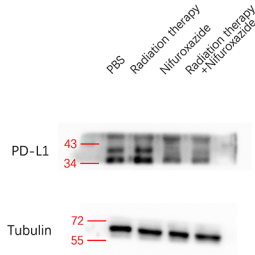

D

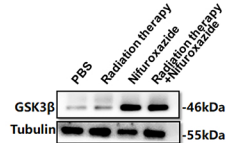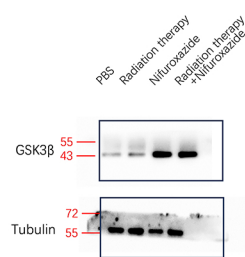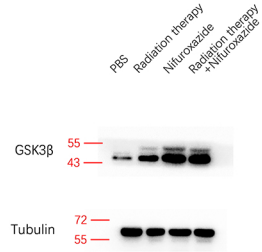

**Figure 8**

Supplement: Figure 8—source data 2. [file elife-90911-fig8-data2.pdf]
