# Supplementary material for: Nifuroxazide suppresses PD-L1 expression and enhances the efficacy of radiotherapy in hepatocellular carcinoma
Source: eLife. 2024 Mar 5;12:RP90911. doi: 10.7554/eLife.90911 (PMC10942647; doi:10.7554/eLife.90911)
Supplement: Supplementary file 2. [file elife-90911-supp2.docx]

| **Name** | **company** | **catalogue number** | **dilution ratio** |
| --- | --- | --- | --- |
| Tubulin | Sigma | T8203 | 1:1000 |
| PD-L1 | bioworld | BS6850 | 1:1000 |
| MMP2 | Cell Signaling Technology | 87809S | 1:1000 |
| Stat3 | bioworld | AP0365 | 1:1000 |
| p-Stat3 | Cell Signaling Technology | 9145S | 1:1000 |
| Pro-caspase3 | Abways | CY5051 | 1:2000 |
| cleaved-caspase3 | Cell Signaling Technology | 9661S | 1:1000 |
| Ki67 | bioworld | BS6667 | 1:1000 |
| PCNA | SANTA | sc-25280 | 1:1000 |
| GSK3β | Abways | CY2434 | 1:2000 |
| cyclin D1 | SANTA | sc-56302 | 1:2000 |
| Bcl-2 | Abways | CY5032 | 1:2000 |
| Bax | Abways | CY5059 | 1:2000 |
| Cytochrome C | BIOPPLE | CY5734 | 1:1000 |
| Pro- caspase9 | Abways | CY5782 | 1:2000 |
| cleaved-caspase9 | Abways | CY5682 | 1:2000 |
| PARP | Cell Signaling Technology | 9542S | 1:1000 |
| cleaved-PARP | Cell Signaling Technology | 5625S | 1:1000 |

**primary antibody：**

**secondary antibody：**

| **Name** | **company** | **catalogue number** | **dilution ratio** |
| --- | --- | --- | --- |
| Horseradish enzyme labeled goat anti rabbit IgG（H+L） | ZSGB-BIO | ZB-2301 | 1:5000 |
| Horseradish enzyme labeled goat anti mouse IgG（H+L） | ZSGB-BIO | ZB-2305 | 1:5000 |
